# Vitamin D deficiency in dengue fever patients' coinfected with *H. pylori* in Pakistan. A case-control study

**DOI:** 10.3389/fpubh.2022.1035560

**Published:** 2022-10-31

**Authors:** Wajid Ameen Mirza, Ke Zhang, Rongguang Zhang, Guangcai Duan, Muhammad Shahid Nawaz Khan, Peng Ni

**Affiliations:** ^1^Department of Epidemiology, College of Public Health, Zhengzhou University, Zhengzhou, China; ^2^The First Affiliated Hospital and International College of Public Health and One Health, Hainan Medical University, Haikou, China; ^3^Department of Medicine, Nishtar Medical University and Hospital, Multan, Pakistan

**Keywords:** vitamin D, dengue fever, *Helicobacter pylori*, coinfection, case-control study

## Abstract

**Introduction:**

Dengue fever is a vector-borne disease with an estimate of 390 million persons getting the infection each year with a significant public health impact. It has been reported DENV patients with vitamin D deficiency led to severe form of dengue infection; while *H. pylori* coinfection alters vitamin D receptors leading to vitamin D deficiency. We hypothesize that DENV patient's having low vitamin D along with *H. pylori* coinfection could have worsen dengue severity as well as vitamin D deficiency. In this case-control study, we compared (I) the vitamin D deficiency in dengue fever cases with or without *H. pylori* coinfection, and (II) negative dengue fever as a control with or without *H. pylori* coinfection. We have also assessed the correlation between vitamin D levels and its effect on warning signs of the dengue fever. Further, we have investigated whether coinfection with *H. pylori* has any effect on warning signs in the dengue fever patients and the vitamin D deficiency in all serotypes of the dengue virus infected patients.

**Methods:**

In this case control study the association of the vitamin D levels with age, gender and *H. pylori* coinfection in dengue fever hospitalized patients was assessed using chi-square and multivariate logistic regression analysis.

**Results:**

Four hundred dengue fever patients with *H. pylori* coinfection were compared with 400 dengue negative controls with *H. pylori* coinfection. The mean age was 29.96 ± 10.5 and 29.88 ± 10.7 years among cases and controls, respectively. Most dengue fever patients with *H. pylori* coinfection were deficient in vitamin D compared with negative dengue controls with *H. pylori* coinfection. In multivariate logistic regression, the dengue cases with *H. pylori* coinfection were.056 times (95% CI: 0.024, 0.128, *P* = 0.000) more likely to have vitamin D “deficiency', while compared with the cases who did not have *H. pylori* coinfection.

**Conclusion:**

The present study proposes that vitamin D deficiency in dengue fever patients coinfected with *H. pylori* is much higher than the dengue fever negative controls coinfected with *H. pylori*. As hypothesized the DENV patient with *H. pylori* coinfection has vitamin D deficiency as well as increased dengue severity.

## Introduction

An estimated 390 million persons are infected with Dengue virus (DENV) each year, and America alone accounts for 14% of cases (1). Also, half of the world's population lives in tropical areas where dengue fever can spread, and the load of the disease is disproportionately higher in low-income populations (2). The *Aedes aegypti* mosquito is the primary vector of the four unique DENV serotypes, and inoculation can result in a variety of clinical manifestations like aches and pain (joint, eye, and muscle pain), rash, nausea, and vomiting. Numerous DENV infections are either asymptomatic or may not necessitate medical attention (subclinical). Acute fever is the clinical emblem of evident or symptomatic dengue, which is further categorized by severity as dengue without warning symptoms (DNWS), dengue with warning signs (DWWS), and severe dengue (SD) (3).

A total of 96 million instances are clinically ostensible each year, resulting in 20,000 deaths due to organ damage, shock, plasma leakage, and hemorrhage (1). Prior infection of dengue virus with a heterologous serotype (4, 5), as well as the immune response of the host, affects more severe clinical outcomes and representations of dengue virus infection (6). Despite the fact that a dengue vaccine candidate, Dengvaxia, has been licensed in over 20 countries, worries about higher risks for seronegative individuals have led to vaccine hesitancy, defined as the delay in accepting or refusing vaccines despite the availability of immunization outset (7, 8). Given the difficulties of administering the dengue vaccine and the lack of a specific therapy, researchers have been looking for biomarkers to predict DENV infection outcomes for a long time. The capacity to discover and manipulate such biomarkers could lead to new therapeutic options for DENV infection severity progression. Micronutrients are intertwined with the immunological response and infection of the host. Because the acute-phase reaction associated with acute viral infection momentarily affects circulation quantities of some nutrients, like vitamin A and iron, that's why the precise measurement of these micronutrients during infection is impossible (8, 9). Acute-phase protein concentrations in the blood can be used to modify these measures (10, 11). Some micronutrient deficits compromise the host immune system and are linked to worse infection outcomes. Iron deficiency affects phagocyte function and T-cell proliferation, and cytokine actions of all phases of the pathogenesis (12, 13). In response to some viruses, a lack of vitamin A lowers the number of phagocytes and weakens cell-mediated immunity (14). Measles, diarrheal infections, and anemia are also more common in people who don't get enough vitamins A (15). On the other hand, macrophage maturation and phagocytosis, the synthesis of pro-inflammatory cytokines, and cell-mediated immunity are all impaired by vitamin D deprivation (16–18).

Vitamin D is essential for child and adult health. Vitamin D insufficiency is frequent in older individuals. The cause could be debilitated cutaneous previtamin D synthesis, lessened solar exposure, reduced renal hydroxylation, inadequate food intake, and residence in a nursing home, paucity of mobility outside the house, dark skin, obesity, and the existence of malabsorption and osteoporosis place elderly individuals at a high risk for vitamin D deficiency and insufficiency. Despite the lack of unanimity regarding the ideal amount of blood 25-hydroxyvitamin D [25(OH) vitamin D], levels below 20 ng/mL are generally regarded just as insufficient vitamin D (19, 20). Studies have also demonstrated that *H. pylori* coinfection alters the tissue and cellular levels of vitamin D receptors. Vitamin D balances the metabolism of calcium and phosphorous, which are imperative for bone development. In addition to its wellknown involvement in bone production, vitamin D also functions as an immunomodulator, targeting numerous immune cells such as monocytes, macrophages, dendritic cells, T-lymphocytes, and B-lymphocytes (21). As a result, vitamin D deficiency may raise the risk of immune system problems and be a risk factor for infection amelioration (22).

Many studies investigated the association of DENV infection and malnutrition (23). Few other studies demonstrated the relationship between *H. pylori* coinfection and vitamin D deficiency and insufficiency. In some studies, the relationship between dengue fever and vitamin D has been studied. The results of these studies showed that the patients with dengue fever had low vitamin D levels, and the deficiency of vitamin D led to SD (24). Also, one of the Meta-analyses (consisting of 48 studies) demonstrated the association of the vitamins (e.g., vitamin D) and *H. pylori*. The results of this Meta-analysis suggested that the *H. pylori* positive patients had lower serum vitamin D levels than *H. pylori* negative patients (25). Both the factors, i.e., Dengue fever and *H. pylori*, lower the vitamin D levels in patients. All of these studies conducted the research on dengue fever and vitamin D deficiency or the *H. pylori* and vitamin D deficiency; none of the studies conducted the research on vitamin D deficiency in dengue fever patients coinfected with *H. pylori*. However, the data on the vitamin D deficiency in DENV patients, coinfected with *H. pylori* is scarce. So here we hypothesize that DENV patients have vitamin D deficiency and coinfection with *H. pylori* even worsen the vitamin D deficiency as well as increased dengue severity. Therefore we have conducted a case-control study to assess the vitamin D deficiency in dengue patients without warning signs (DNWS) and dengue patients with warning signs (DWWS) coinfected with *H. pylori*. Thus, to fill the gap, in this case-control study, we compared vitamin D levels among dengue fever cases, either *H. pylori* + or *H. pylori—*with healthy controls. We have also assessed the relationship between vitamin D levels and its effect on DNWS and DWWS patients. We have further investigated whether coinfection with *H. pylori* has any effect on the sign and symptoms of the dengue fever patients. We have also assessed the vitamin D deficiency in all serotypes of the dengue virus.

## Methods

### Study design

A case-control study of 400 hospitalized patients with dengue fever was carried out in a single center in Pakistan from July 2020 to January 2021. The Nishtar Medical University and Hospital is a public university of the health sciences in Pakistan. It is situated in the city of Multan in the province of Punjab. Tertiary care is provided at Nishtar Hospital, which serves a significant portion of the population of South Punjab as well as parts of the neighboring provinces of Baluchistan and Khyber Pakhtunkhwa. The present overall bed capacity of the hospital is 1,800, making it one of the largest hospitals in the country. In our study, patients between the ages of 13 and 80 with a doctor-diagnosed DENV and either *H. pylori* + or *H. pylori*—coinfection were admitted for in-ward care over the course of seven months were included. Information about socioeconomic status, medical history, test results, and warning signs were gathered during the patient enrollment process. The hematocrit, platelet count, and other laboratory indicators were determined after a physical examination. It was decided to exclude those patients who had been previously diagnosed with or had any history of diabetes or AIDS, as well as those with hematological or cancerous illnesses or cardiac disease. We also excluded those patients who had severe bleeding, hypoalbuminemia, effusions, or shock at baseline. To compare vitamin D levels, we recruited 400 control participants from the same hospital. As a result, the controls were matched based on gender and age to those in the cases. Controls with a chronic sickness, bone abnormalities, or a known history of dengue fever were excluded. All of the controls were unrelated and lived in and around the region of Multan. Both the cases and the controls were Punjabi speakers from Pakistan's South Punjab region.

### Diagnosis of DENV, *H. pylori*, and vitamin D

Warning signs, as well as daily microhematocrit measurements, were gathered. Patients' platelet counts were measured on a daily basis. Serum samples were transferred to the Nishtar hospital's virology lab for storage at−70°C. Using previously reported procedures, samples were analyzed for dengue virus infection using a diagnosis algorithm that included IgM enzyme-linked immunosorbent assay (ELISA) in matched sera or viral isolation, NS1 antigen detection, or RT-PCR in acute samples (26). ELISA was used to identify dengue IgM antibodies in a patient's convalescent blood 7–15 days after the onset of symptoms. Viral isolation, positive NS1 antigen in acute serum, a shift from negative to positive IgM test results for dengue virus antibodies, or a fourfold increase in previously existing dengue virus antibody levels were all considered to be signs of dengue infection.

For the detection of *H. pylori* both in cases and controls, we used a breath test. After an overnight fast, a 13C-UBT utilizing the Proto Pylori kit (Isodiagnostika, Canada) containing 75 mg of 13C-urea and other additives were conducted. To assess the two breaths samples taken 30 minutes apart we used the isotope/gas chromatography ratio mass spectrometry.

By explaining the purpose of the study and making sure the anonymity and confidentiality of the data written informed consent were obtained from all the patients. Vitamin D deficiency has been historically defined and recently recommended by the Institute of Medicine (IOM) as a 25(OH) D less than 20 ng/ml. The “insufficient” level of vitamin D has been defined as a 25(OH) D of 21–29 ng/ml (19, 27–32). To detect the vitamin D, a 3 ml sample of venous blood was obtained from every patient while observing all the aseptic measures. To analyze the total serum 25(OH) D, we used the Liaison 25 (OH) vitamin D Total Assay kit (DiaSorin Inc. USA) in batches; the results of this analysis were not available for the management of the patients. Total circulating 25(OH) D [25(OH) D2 and 25(OH) D3] was considered as the acceptable biomarker for serum vitamin D status (33). As per manufacturer instructions, the analytical range of the kit was 4–150 ng/ml, and it also has an acceptable interassay and intra-assay variability.

### Dengue virus serotypes

We used QIAGEN QIAamp viral RNA mini kit (QIAGEN, Hilden, Germany) on all serologically positive samples of the blood to extract the RNA, as per manufacturer instructions. The extracted RNA was then adapted for the identification of dengue virus serotypes (1–4), using type specific RT-PCR, as purported earlier (34, 35).

### Liver enzymes profile

Valuation of the liver enzyme profile was conducted on all serial samples of the blood on a daily basis. We used two tests to assess the damage of the liver cells, 1; aspartate aminotransferase (AST), 2; alanine transaminase (ALT). To assess the intrahepatic and hepatobiliary cholestasis, the alkaline phosphatase (ALP) test was used by using an automated biochemical analyzer (Thermo scientific clinical chemistry analyzer, USA).

### Statistical analysis

For data analysis, we used version 22 of the Statistical Package for the Social Sciences (SPSS). The normality of data was also tested. Descriptive statistics are expressed as frequency, percentages, mean ± SD, as necessary. Chi-square test and Mann-Whitney U test were applied for the comparison of the cases and control. For comparison of vitamin D levels in all serotypes of the dengue virus a chi square test was used. Multivariate logistic regression analysis was used for the associated factors of the vitamin D deficiency and insufficiency. We used vitamin D level as categorical variable for this analysis. The categories of the vitamin D used were “deficient” < 20 ng/ml, “insufficient” ≥20 ng/ml, < 30 ng/ml, and “normal” ≥30 ng/ml. These values were picked using wellestablished definitions of vitamin D status (36). Two-tailed *P*-value of < 0.05 was considered as statistically significant.

## Results

A total of 550 patients with dengue fever were identified. One hundred and forty patients were excluded from the study, and the data from the 10 patients could not be collected as they were transferred out. A reason to dropout was the denial of the consent. Four hundred healthy patients were included as controls. Baseline characteristics of cases and controls are given in Table 1. Four hundred dengue fever (DNWS and DWWS) cases coinfected with *H. pylori* + or *H. pylori*—were compared with 400 healthy controls. All the controls were serologically negative for dengue virus. Mean age was 29.96 ± 10.5 and 29.88 ± 10.7 years among cases and controls, respectively. No significant difference was found in the age and gender of the cases and the controls. However, the vitamin D “deficiency” (< 20 ng/ml) in dengue fever cases with *H. pylori* coinfection was much higher (*P* = 0.000) as compared to controls with *H. pylori* coinfection. Mean vitamin D level was 18.8 ± 12 ng/ml and 54.6 ± 22 ng/ml in cases and controls, respectively. The mean Vitamin D levels showed a significant difference among cases and controls. A comparison of the vitamin D “deficiency”, “insufficiency” and “normal” levels was carried out between cases of either *H. pylori* positive or negative and controls either *H. pylori* positive or negative (Table 1).

**Table 1:** Baseline characteristics and vitamin D levels of cases and controls.

**Characteristics**	**Cases (400)**			**Controls (400)**		
	***H. pylori +***	***H. pylori -***	***P-value***	***H. pylori +***	***H. pylori -***	***P-value***
Age (years)			0.78			0.55
Youth (13–24)	120	20		112	32	
Adults (25–60)	221	36		199	53	
Seniors (>60)	3	0		4	0	
Gender			0.52			0.14
Male	249	41		224	66	
Female	95	15		91	19	
Serum vitamin D			0.000			0.51
Deficient (< 20 ng/ml)	255	24		16	3	
Insufficient(≥ 20– < 30 ng/ml)	77	12		33	6	
Normal(≥30 ng/ml)	12	20		266	76	
ALT (normal)	99	17	0.46			
AST (normal)	6	4	0.03			
Alkaline phosphatase	307	49	0.66			
Hematocrit	92	12	0.70			
Platelets	149	23	0.48			

For comparisons, ANOVA, the chi-squared test, and the Mann–Whitney *U* test were used as appropriate.

While looking at the results in Table 1, it is obvious that among cases with *H. pylori* coinfection, the frequency of the vitamin D “normal” level 12 (3%) was significantly lower as compared to the controls 266 (66.5%) with *H. pylori* coinfection. During dengue infection, the eminence of liver transaminases crops up, so we paged to investigate the levels of the liver enzymes. Both ALT 125 ± 134 and ALP 102 ± 60 elevated during the course of the dengue illness, but the difference in *H. pylori* + and *H. pylori*—groups, did not reach the significance level. Also the difference in the hematocrit 43 ± 6 and platelet 44 ± 37 values was not significant in both groups. Apart from the liver, ALP also originates from many tissues such as the placenta, intestine, and bones (37). The elevation of the ALP occurs in hepatobiliary diseases that emanate in cholestasis (37). Though 34 patients from *H. pylori* + group and only 7 patients from *H. pylori*—group had a mild altitude of ALP levels, nearly all other patients throughout the duration of the dengue illness had normal ALP levels, which exhort that cholestasis is improbable to occur in dengue lured liver disease. On the other hand, the level of the AST 249 ± 376 was much more elevated in the *H. pylori* + group as compared to the *H. pylori—*group (*P* = 0.03).

There was no significant difference in the dengue illness's warning signs, i.e., persistent vomiting, shortness of breath, giddiness, lethargy, gum bleed, or headache among *H. pylori* + and *H. pylori*—groups, as shown in Table 2. While the other warning signs such as rash (*P* = 0.03), worsening clinical signs (*P* = 0.01), abdominal pain (*P* = 0.02), and diarrhea (*P* = 0.02) were more severe in dengue fever cases with *H. pylori* coinfection as compared to cases that do not have *H. pylori* coinfection.

**Table 2:** Warning signs of dengue fever in *H. pylori* + and *H. pylori—*groups.

**Warning signs**	**Cases (400)**		***P*-value**
	***H. pylori* + n (%)**	***H. pylori—*n (%)**	
Persistent vomiting	151 (84.4%)	28 (15.6%)	0.23
Rash	145 (90.1%)	16 (9.9%)	0.03
Worsening clinical signs	81 (93.1%)	6 (6.9%)	0.01
Shortness of breath	47 (90.4%)	5 (9.6%)	0.22
Giddiness	33 (86.8%)	5 (13.2%)	0.55
Lethargy	105 (88.2%)	14 (11.8%)	0.25
Abdominal pain	72 (93.5%)	5 (6.5%)	0.02
Gum bleed	17 (94.4%)	1 (5.6%)	0.25
Diarrhea	72 (93.5%)	5 (6.5%)	0.02
Headache	79 (88.8%)	10 (11.2%)	0.25

**Figure 1:**
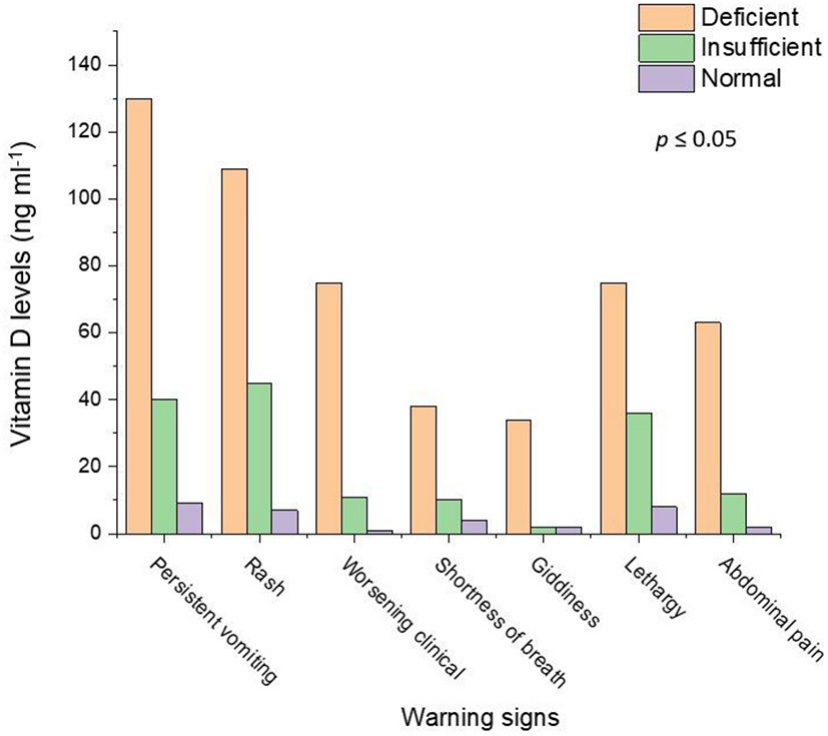
Relationship of the dengue patients with warning signs and vitamin D levels. Light salmon color represents vitamin D deficiency, Light green color shows vitamin D insufficiency and violet color depicts the normal level of the vitamin D.

**Figure 2:**
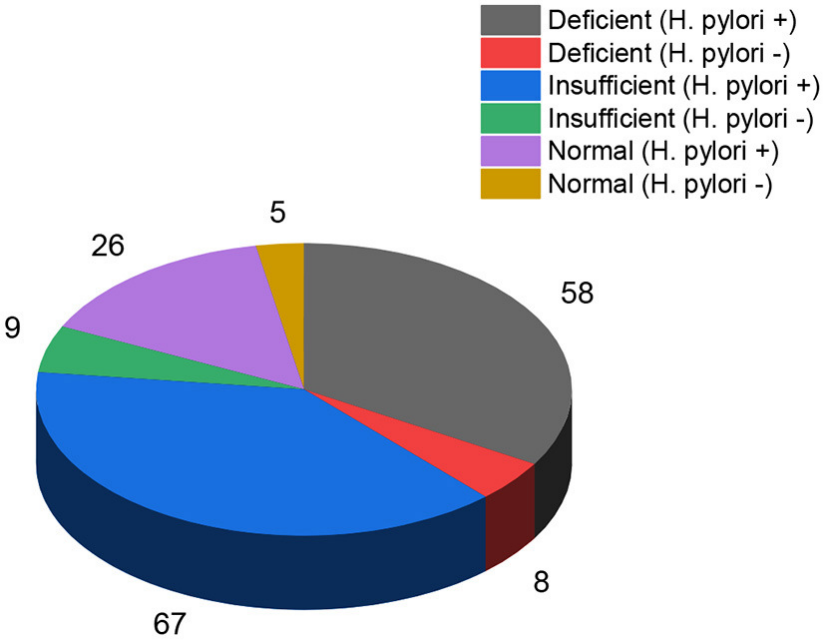
“Deficient”, “insufficient” and “normal” levels of vitamin D in dengue fever (*H. pylori* + and *H. pylori*-) patients without warning signs.

Figure 1 shows the relationship between the warning signs of the dengue illness and vitamin D levels. The dengue patients who were “deficient” in vitamin D had more severe warning signs. There were 72.6% of dengue patients with persistent vomiting who were also “deficient” in vitamin D. The percentage of the worsening clinical signs (heart rate and fluctuated blood pressure) (86.2%) were higher in dengue patients with “deficient” vitamin D levels. The severity of the other warning signs such as giddiness (89.5%), lethargy (63%), abdominal pain (81.8%), and diarrhea (84.4%) was seen in the dengue patients with “deficient” vitamin D levels. In our study 400 total cases were admitted for in ward care. Out of 400, 43% (173 patients) were admitted with DNWS (Figure 2), and, 227 (57%) patients were admitted with DWWS. Conclusively, all the patients 142 (“deficient” and “insufficient” vitamin D levels) of DNWS were progressed to DWWS but 31 patients with ‘'normal” vitamin D level were not progressed to SD. Warning signs of progression to SD usually occur in the late febrile phase around the time of defervescence, and include persistent vomiting, rash, shortness of breath, giddiness, lethargy, abdominal pain, gum bleed, diarrhea and, headache (38). In the current study from the total 173 patients of DNWS, 66 (*H. pylori* +, 58 and *H. pylori* –, 8) were “deficient” in vitamin D. While 76 (*H. pylori* +, 67 and *H. pylori* –, 9) were “insufficient” in vitamin D. The “normal” level of vitamin D was present only in 31 (*H. pylori* +, 26 and *H. pylori* –, 5) patients.

**Figure 3:**
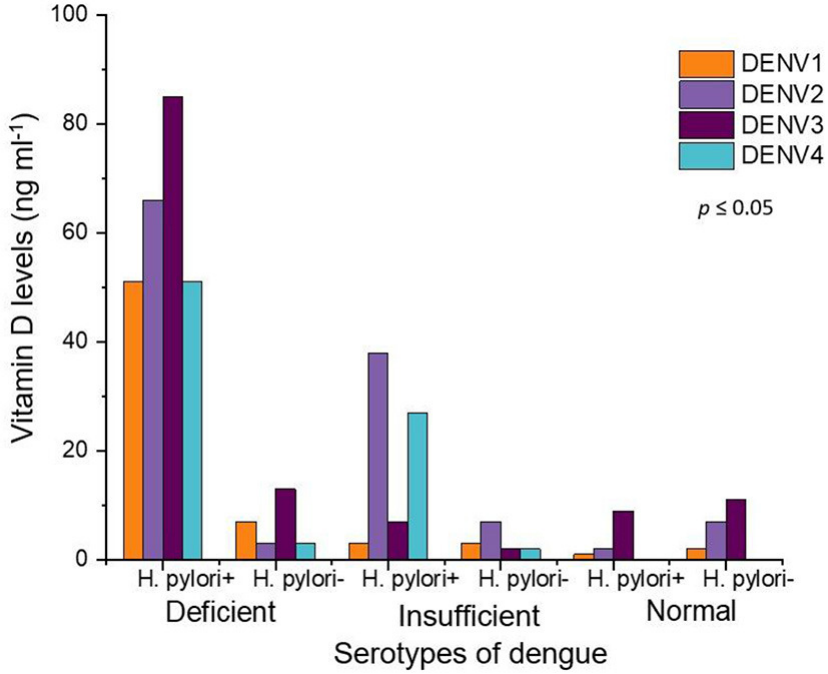
Vitamin D levels in all serotypes of dengue fever patients. Orange color represents DENV 1, purple shows DENV 2 while maroon DENV 3 and turquoise depict DENV 4 serotypes.

In our study, the cases were infected with all four serotypes (DENV 1, DENV 2, DENV 3, and DENV 4) of the dengue virus. The numbers of cases in DENV 1 serotype were 67, (*H. pylori* +, 55 and *H. pylori* –, 12). The numbers of cases in DENV 2 serotype were 123, (*H. pylori* +, 106 and *H. pylori*–, 17). The numbers of cases in DENV 3 serotype were 127, (*H. pylori* +, 101 and *H. pylori*-, 26) and the numbers of cases in DENV 4 serotype were 83, (*H. pylori* +, 78 and *H. pylori* –, 5). The most “deficient” level of vitamin D was seen among the cases infected with DENV 3 (*H. pylori* +, 85 and *H. pylori* –, 13) followed by DENV 2 (*H. pylori* +, 66 and *H. pylori* –, 3), DENV 1 (*H. pylori* +, 51 and *H. pylori -*, 7*)*, and DENV 4 (*H. pylori* +, 51 and *H. pylori* –, 3), respectively, Figure 3. On the other hand, the cases infected with DENV 2 (*H. pylori* +, 38 and *H. pylori* –, 7), had the most “insufficient” level of vitamin D. There were few cases in the “normal” category of vitamin D levels in DENV 1 (*H. pylori* +, 1 and *H. pylori* –, 2), DENV 2 (*H. pylori* +, 2 and *H. pylori* –, 7), DENV 3 (*H. pylori* +, 9 and *H. pylori* –, 11), While there were no cases, in the “normal” vitamin D level category, infected with DENV 4.

### Multivariate logistic regression analysis

To determine the co-related factors for vitamin D “deficiency” and “insufficiency,” a multivariate logistic regression analysis was performed. The results suggested that the dengue cases with *H. pylori* coinfection were 0.056 times (95% CI: 0.024, 0.128, *P* = 0.000) more likely to have vitamin D “deficiency”, while compared them with the cases who did not have *H. pylori* coinfection. On the other hand, the dengue cases coinfected with *H. pylori* were 0.092 times (95% CI: 0.035, 0.236, *P* = 0.000) more likely to have vitamin D “insufficiency” while compared them with cases who did not have *H. pylori* coinfection as shown in Table 3.

**Table 3:** Models derived with multivariate logistic regression analysis of associated factors of vitamin D deficiency and insufficiency.

**Independent variables**	**β**	**Odds ratio (95% CI)**	***P***
**Vitamin D deficiency**			
Age	0.256	1.29 (0.568, 2.93)	0.54
Gender	−0.462	0.63 (0.26, 1.50)	0.29
*H. pylori* coinfection	−2.89	0.05 (0.02,0.12)	0.00
**Vitamin D insufficiency**			
Age	0.58	1.8 (0.73, 4.3)	0.19
Gender	−0.57	0.56 (0.21, 1.45)	0.23
*H. pylori* coinfection	−2.39	0.09 (0.03,0.23)	0.00

## Discussion

In this case-control study, we compared the vitamin D levels in the DNWS and DWWS cases coinfected with *H. pylori* with dengue fever negative controls coinfected with *H. pylori*. Vitamin D deficiency was more prevalent in the youth and seniors age patients, and the situation was even worse in the cases with *H. pylori* coinfection. The possible explanation of vitamin D deficiency in dengue fever patients could be interpreted in many ways. In response to increased viral loads due to SD, a high concentration of the cytokine by T cells, macrophages, endothelial cells, and monocytes is released (39). Vitamin D helps in restricting the viral replication by supporting macrophages differentiation. Also, the dengue infected macrophages treated with vitamin D produce significantly less cytokine, as compared to the macrophages that do not receive vitamin D treatment (40). The literature available on the affirmation of the co-relation of vitamin D level and dengue infection is sparse and inconsistent (41, 42). Another study demonstrated the low vitamin D level among adults with dengue infection as compared to unmatched dengue negative healthy controls. The sample included in the study was a case-to-control ratio of 1:1. Spectrophotometer method was used to analyze the level of the vitamin D. It is imperative to keep in mind that laboratory techniques and methods used may lead to systematic errors, and this could cause inconstancy of research findings. A recent study in India demonstrated contrasting results (42, 43).

Another study demonstrated the association of the febrile phase, vitamin D level, and its relationship with the advancement of the dengue illness to SD in adults and children. The results of this study stated that the low serum levels of the vitamin D during the early febrile phase anticipate the decreased odds of progression to SD infection. Comprehensively, these disparities in different studies may be justified by the discrepancy in the disease severity and duration, age of the patients, DENV serotypes, and case definitions (43).

In our study all the DNWS with “deficient” and “insufficient” levels of vitamin D and *H. pylori* coinfection were progressed to DWWS. Our results are supported by a recent study conducted in Singapore (44) in which low systemic 25-(OH) D was associated with SD. Another study (41) reported the same results in which there was low vitamin D status among adults with DWWS compared to unmatched healthy controls. The sample included a case-to-control ratio of 1:1 (15 subjects in each group), and vitamin D level was analyzed using a spectrophotometric method. While our study results are in contrast with another study (43). This study concluded the odds of progression from DNWS to SD increase when patients had low serum i.e., 25(OH) D concentration. Vitamin D is also known to suppress Th1 cytokines and enhance IL-10 production by peripheral blood mononuclear cells in response to microbial antigens (45–47). Since IL-10 is known to play a role in dengue disease pathogenesis (48), it is possible that vitamin D could also contribute to disease pathogenesis through altered IL-10 response. Since the effect of vitamin D is also dependent on single nucleotide polymorphisms in the VDR (vitamin D receptor) gene (49), the influence of vitamin D on dengue in the context of host genetics needs to be investigated.

In our study, we have also investigated the involvement of the liver; we have found that the dengue patients with *H. pylori* coinfection had some degree of the involvement of the liver. After the liver cell injury, the two indicators ALT and AST are released into circulation. Despite the fact that in low concentrations, the ALT is also found in intestine tissues, brain and skeletal muscles, but it is principally contemplated as a specific liver enzyme (50). On the other hand, AST is released in circulation after injury to liver cells, skeletal and cardiac muscles (51). In our study, we found that the increase in the AST was more obtrusive than the rise in ALT levels, in patients with *H. pylori* coinfection. This indicates the damage to the other organs of the body aside from the liver, which could be a contributory factor of the rise in the serum ALT level. We have also measured the modulation in alkaline phosphatase (ALP). Despite the fact that the ALP is one of the markers of cholestasis, it can be exalted due to oxidative stress. In dengue fever there is an increase in the oxidative stress, which is associated with the severity of the illness (52, 53). In our study there were no patients who had significant levitation in ALP levels which proposes that there was minimum cholestasis in the dengue fever patients coinfected with *H. pylori*.

The results of the preclinical studies disclosed the biological mechanism of vitamin D and its ability to modulate the immune system. The physiological effects of the vitamin D are wielded by intracellular vitamin D receptors. These receptors are conveyed by every nucleated cell in the body, including B lymphocytes, antigen presenting dendritic cells, T lymphocytes, and macrophages (54). The receptors of the vitamin D activate gene expressions of antimicrobial peptides with the help of epithelial cells, monocytes, and neutrophils. There are two important antimicrobial peptides which include cathelicidin and β-defensin (55). Cathelicidins have antimicrobial effects against viruses, parasites, gram-positive bacteria, fungi, and gram negative bacteria (56). The role of the active form of the vitamin D, 1, 25-dihydroxyvitamin D is to enhance the cathelicidin expression in *H. pylori* infected gastric epithelial cells; in this way, the vitamin D has a very crucial semblance in regulation of mucosal immunity against the *H. pylori* (57). The other β-defensin antimicrobial peptide is also secreted from the gastric epithelium infected with *H. pylori* and wields anti-bacterial effects on the mucosal surface (58). The deficiency of the vitamin D may coerce to lower the mucosal immunity due to diminished cathelicidin and β-defensin secretion; thus, the host may fail to eliminate *H. pylori*. This could at least partly describe the elevated *H. pylori* coinfection prevalence in the patients with low vitamin D levels.

For longer than a century, the association between vitamin D deficiency and perceptivity to infection has been implied, with the earliest observations that the children were more likely to have respiratory infections if they experience nutritional rickets, this led to the phrase of “rachitic lung” (59). A latest study describes that vitamin D exerts an antimicrobial effect on *H. pylori*. In this study it is found that vitamin D has a very crucial part to play in the homeostasis of the mucosa and protection of the host from the *H. pylori* coinfection (57). Apart from the effects of the vitamin D on bone metabolism, it may reduce the inflammatory markers such as IL-18, CRP, IL-6, and TNF-α, and the level of the anti inflammatory cytokine IL-10, may increase (60). Vitamin D regulates the expression of AMPs cathelicidin and β-defensin, which kill the bacteria. Even though the effect of the cathelicidin has only seen in macrophages infected with *Mycobacterium tuberculosis*, the antimicrobial effect of the cathelicidin against gram positive and gram negative bacteria have also been reported (55). In a vitamin D deficiency state, the infected macrophages are incapable to synthesize ample amount of 1, 25-(OH) D2 to upregulate the production of cathelicidin and β-defensin, thus apprehending them ineffectual to kill the *H. pylori*.

In another case-control study, (39) the likelihood of having vitamin D deficiency was higher among children with SD compared to healthy controls, these findings are consistent with the results of our study. We have found that the vitamin D deficiency is higher in dengue fever cases with *H. pylori* coinfection as compared to the controls. The possible association of vitamin D deficiency with SD could be explained in several ways. First, it is known that severe dengue is linked to high cytokine concentrations released by T cells, monocytes/macrophages, and endothelial cells in response to high viral loads. Second, vitamin D supports macrophage differentiation, thus restricting viral replication. Also, it has been shown that cytokine production is significantly lower in dengue infected macrophages with vitamin D treatment than that without (40).

The immune mechanisms for observations of 25-(OH) D association with dengue disease course and severity are not entirely elucidated. Few authors have evaluated this in more detail. Of interest, an *in vitro* study involving human myelomonocytic and hepatic cell lines exposed to various concentrations of 1,25-(OH)2 D3 which were subsequently infected with DENV 4 found significantly reduced percentage of infected cells. In our study the patients with reduced vitamin D levels (“deficient” and “insufficient”) were inclined to infection with DENV 4 serotype. The possible mechanism could be reduced production of TNF α, IL-1B, IL-6, IL-12p70 with a dose-response relationship observed with 1,25-(OH)2 D3 (61). The underlying immune mechanisms are not yet clear. Arboleda Alzate et al. (62) exposed monocytes-derived macrophages (from healthy volunteers) *in vitro* to varying concentrations of 1,25-(OH)2D3 with subsequent infection with DENV 2. The macrophages differentiated in the presence of higher 1,25-(OH)2D3 concentrations had decreased DENV 2 infectivity, potentially due to reduced expression of receptors required for DENV entry into macrophages and also had reduced pro-inflammatory cytokine levels (specifically TNF α, IL-1β, IL-10) in response to DENV infection (62). Another *in vitro* study challenged monocytes-derived macrophages from participants enrolled in a vitamin D supplementation study with DENV 2. Macrophages from participants exposed to higher-dose (4,000 IU/day) supplementation were not as susceptible to DENV 2 infection compared to those who received lower dose supplementation, thereby having a protective effect with TNF-α level were lower while IL-10 and IL-8 were higher in the higher dose supplementation group (63). However, serum 25-(OH) D levels were not quantified in this study. Similar results are found in our study, in which more dengue fever patients are infected with DENV 2 serotype having “deficient” and “insufficient” vitamin D levels. There are many immunological postulations as to how vitamin D may be influencing the susceptibility to infection and inflammatory response; however this still needs further study.

In our study, we have also investigated the vitamin D levels in all four serotypes of the dengue virus in cases coinfected with *H. pylori*. Most of the cases that were not coinfected with *H. pylori* have “normal” vitamin D levels. The patients having dengue virus serotype 3 and coinfected with *H. pylori* have the most “deficient” vitamin D levels. While in, the patients having dengue virus 2 serotype and also coinfection of *H. pylori* have the most “Insufficient” vitamin D levels. These results demonstrate that dengue virus infection, along with *H. pylori* coinfection affects the vitamin D levels badly. This also indicates an association between the dengue virus serotypes, *H. pylori* coinfection, and vitamin D levels. Moreover, the dengue fever patients coinfected with *H. pylori* had severe warning signs like rash, abdominal pain, and diarrhea as compared to dengue fever patients that were not coinfected with *H. pylori*. Generally, it has been observed that colonization by *H. pylori* causes a strong systemic immune response, creating a chronically inflamed environment with reduced stomach acidity that favors the growth of other bacteria in the gastric environment, maintaining the inflammation and thereby reducing the level of vitamin C in the gastric juice (64). One widely accepted hallmark of *H. pylori* is that it successfully and stealthily evades host defense mechanisms. Though the gastric mucosa is well protected against the infection, *H. pylori* are able to reside under the mucus, attach to gastric epithelial cells and cause persistent infection by evading immune responses mediated by the host (65). Although not widely known, *H. pylori* can also affect organ systems outside of the gastrointestinal tract. It is now apparent that *H. pylori* can infect the skin, liver and heart and that these infections may produce a number of different disease states. In addition, *H. pylori* coinfection can adversely affect the nutritional status of both children and adults (66). The presence of *H. pylori* coinfection reduces the body's immune system. In the state of lower body immune response and fight against dengue fever, the patients exhibit more severe sign and symptoms of dengue fever as compared to the patients who had no *H. pylori* coinfection and had a better immune system. To our knowledge, this is the first study to explore the comparison of the vitamin D levels in DNWS and DWWS patients coinfected with *H. pylori* and dengue fever negative controls coinfected with *H. pylori*.

The results of this study should be explained with few limitations. Despite the fact that case-control studies can be used to establish a relationship between exposures and outcomes, these studies cannot be used to establish causation. In our study, cases were compared with healthy controls, beyond documented evidence of dengue infection. The quality of the results of the present study could have been enhanced by taking another group of patients for comparison with SD. We aimed the present case-control study in a single center and commenced a significant relationship between DNWS and DWWS patients coinfected with *H. pylori* and vitamin D deficiency. Further case-control studies could enroll multiple controls to each case to escalate the statistical power of the study. Multicenter research over an extended duration of time would have given more precise and convincing results, as the patients recruited from multicenter are asserted to be the representative. In this study, the cases and controls came from the same catchment population but matching them for confounding factors, environmental variables and area of residence may have probably added on the validity of the results.

## Conclusions

The present study proposes that vitamin D deficiency in dengue fever patients coinfected with *H. pylori* is much higher than the dengue fever negative controls coinfected with *H. pylori*. Large scale multi center studies are critical to know any association of the dengue virus and *H. pylori* coinfection and their role in the deficiency of the vitamin D. It should also be investigated whether engorgement with vitamin D is favorable in the prevention of the severe form of the dengue fever and its warning signs in *H. pylori* coinfected patients. Many studies show that *H. pylori* have a protective effect on asthma (67, 68) and malaria (69). Due to case-control nature of the present study, we could not assess whether *H. pylori* coinfection has any protective effect on all or any specific serotype of the dengue virus.

## Data availability statement

The original contributions presented in the study are included in the article/supplementary material, further inquiries can be directed to the corresponding authors.

## Ethics statement

The studies involving human participants were reviewed and approved by the ethics review committee of the Nishtar hospital and by the institutional review board of Zhengzhou University. Written informed consent to participate in this study was provided by the participants' legal guardian/next of kin.

## Author contributions

Conceptualization: WM and MK. Methodology: KZ and PN. Formal analysis: MK and PN. Writing—original draft preparation: WM. Writing—review and editing: RZ, KZ, GD, and WM. Supervision and project administration: RZ. All authors contributed to the article and approved the submitted version.

## Funding

This study was funded by the National Natural Science Foundation of China-Henan Joint Fund: 81773495.

## Conflict of interest

The authors declare that the research was conducted in the absence of any commercial or financial relationships that could be construed as a potential conflict of interest.

## Publisher's note

All claims expressed in this article are solely those of the authors and do not necessarily represent those of their affiliated organizations, or those of the publisher, the editors and the reviewers. Any product that may be evaluated in this article, or claim that may be made by its manufacturer, is not guaranteed or endorsed by the publisher.
